# Microalgal diversity fosters stable biomass productivity in open ponds treating wastewater

**DOI:** 10.1038/s41598-017-02139-8

**Published:** 2017-05-16

**Authors:** Dae-Hyun Cho, Jung-Woon Choi, Zion Kang, Byung-Hyuk Kim, Hee-Mock Oh, Hee-sik Kim, Rishiram Ramanan

**Affiliations:** 1 Cell Factory Research Center, Korea Research Institute of Bioscience and Biotechnology (KRIBB), Yuseong-gu, Daejeon 305-806 Republic of Korea; 2 Green Chemistry and Environmental Biotechnology, University of Science and Technology (UST), Yuseong-gu, Daejeon 305-350 Republic of Korea; 3 Department of Chemical and Biomolecular Engineering, Korea Advanced Institute of Science and Technology (KAIST), Daejeon, 305-701 Republic of Korea; 4 Department of Environmental Science, School of Earth Science Systems, Central University of Kerala, Kasaragod District, Kerala India

## Abstract

It is established that biodiversity determines productivity of natural ecosystems globally. We have proved that abiotic factors influenced biomass productivity in engineered ecosystems i.e. high rate algal ponds (HRAPs), previously. This study demonstrates that biotic factors, particularly microalgal diversity, play an essential role in maintaining stable biomass productivity in HRAP treating municipal wastewater by mutualistic adaptation to environmental factors. The current study examined data from the second year of a two-year study on HRAP treating municipal wastewater. Microalgal diversity, wastewater characteristics, treatment efficiency and several environmental and meteorological factors were documented. Multivariate statistical analyses reveal that microalgae in uncontrolled HRAPs adapt to adverse environmental conditions by fostering diversity. Subsequently, five dominant microalgal strains by biovolume were isolated, enriched, and optimum conditions for high biomass productivity were ascertained. These laboratory experiments revealed that different microalgal strains dominate in different conditions and a consortium of these diverse taxa help in sustaining the algae community from environmental and predatory pressures. Diversity, niche or seasonal partitioning and mutualistic growth are pertinent in microalgal cultivation or wastewater treatment. Therefore, enrichment of selective species would deprive the collective adaptive ability of the consortium and encourage system vulnerability especially in wastewater treatment.

## Introduction

In natural ecosystems, it was demonstrated that biodiversity mediates biomass productivity of the entire ecosystem^1^. This is particularly noteworthy with respect to primary producers in both terrestrial and aquatic ecosystems. In natural aquatic ecosystems, it has been observed that top-down control has a significant effect on biomass productivity compared to fertilization, which was shown to have null effect^1^. It is also known that biodiversity improves water quality through niche partitioning^2^. However, in engineered ecosystems like wastewater treatment plants such hypotheses have not been well established. In fact, few, unintegrated studies on species richness in algal ponds or microcosm experiments have thrown up diverse conclusions^3, 4^. Therefore, it is pertinent to look at the impact of biodiversity over productivity in engineered ecosystems subjected to natural variations like high rate algal ponds (HRAPs). HRAPs treating wastewater can serve as excellent model systems for testing these hypotheses as it offers heterogeneous environments and avoids over-simplification akin to natural ecosystems. Apart from testing these hypotheses, increasing algal biomass productivity is of commercial importance as a variety of bioproducts are derived from algae^5^.

HRAPs have several advantages over traditional microalgal ponds. These include increased treatment efficiency emanating from better aeration, mixing and less residence time^5, 7, 8^. However, HRAPs treating wastewater are influenced by variety of factors including temperature, variable nutrient concentration and organic loading rates, and microbial diversity of the influent^7, 8^. Our previous study established the role of abiotic factors, particularly temperature and organic carbon, in influencing biomass productivity in HRAPs treating wastewater during the first year (2013–2014)^6^. These factors coupled with other meteorological factors would reduce the overall efficiency of HRAPs and ultimately affect the biomass productivity^6^. The algal biomass productivity is reduced with decrease in inorganic carbon supply, but at the same time, high organic carbon loading rate encourages bacterial growth. Similarly, low N:P ratios vastly affect algal growth compared to cyanobacteria^4^. Under favourable conditions, algae can outcompete bacteria and cyanobacteria in HRAP^7, 9^. Predators also play a major role in controlling the microalgal biomass productivity, at times resulting in system collapse, also termed as crashes^8, 10^. However, for algal biomass production for bioproducts with or without the use of wastewater, consistently high biomass productivity throughout the year is the most desirable trait^10^.

Therefore, it is important to understand the abiotic and biotic factors which determine the microalgal productivity in this engineered ecosystem, analogous to natural ecosystems^8, 10^. We compute various environmental factors affecting biomass productivity and microbial diversity in HRAP for the second consecutive year (2014–2015) and in turn the effect of biotic factors on this engineered ecosystem. This study would also help in ascertaining the role of selective breeding in limiting functional traits^8, 12^. The study establishes the need for multiple functional traits in a challenging habitat like HRAP treating wastewater to sustain biomass productivity under various environmental and predator pressures.

## Results and Discussion

### Biomass productivity in HRAP cultivated in wastewater

**Figure 1:**
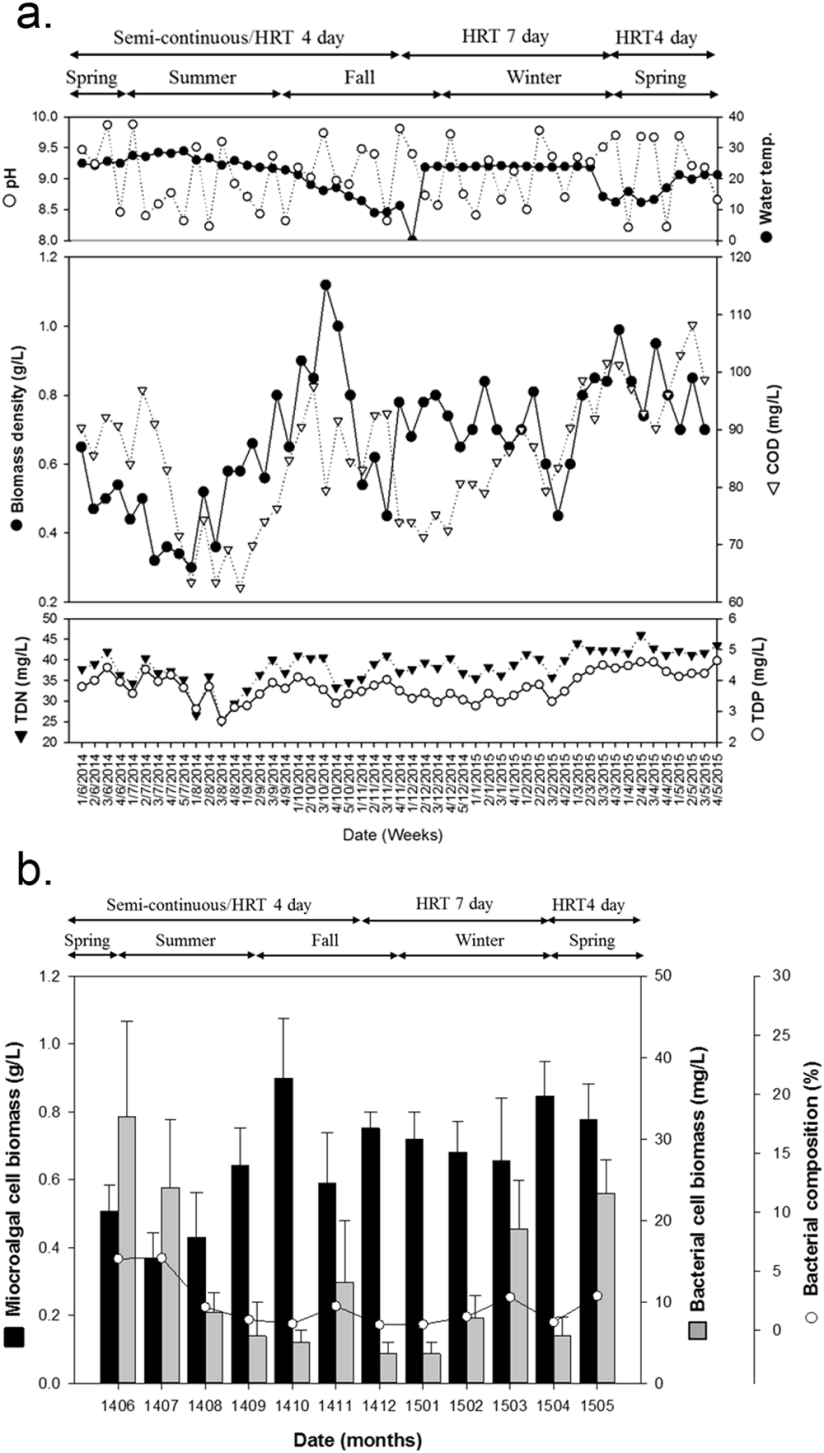
(**a**) Characteristics of influent wastewater in the HRAP for the year 2014–15, featuring all four seasons. (**b**) Microalgal and bacterial biomass in HRAP for one year.

The average physicochemical characteristics of the untreated influent wastewater in the study period were as follows; COD_-Mn_ (Chemical Oxygen Demand, ppm): 158.21 (±10.50), TSS (Total Suspended Soilds, ppm): 133.56 (±18.43), TN (Total Nitrogen, ppm): 42.08 (±3.88), TP (Total Phosphorous, ppm): 4.33 (±0.59). The average N:P ratio of the influent was 10.03 during the study period (Table S1). Average biomass concentration for the second year was 0.7–0.8 g/L. Unlike in the first year, the biomass concentration was relatively stable as the temperature was controlled at 20 °C in winter months (Fig. 1a). As the temperature was constant, we hypothesized that the biomass concentration in the second year (current study period; June 2014–May 2015) might be only influenced by two other factors, viz organic carbon and inflow microbial diversity, as reported earlier^6^. Initial results indicated that COD and nutrient content (N/P) in the influent wastewater had a relatively high influence on biomass concentration (Fig. 1a). Moreover, heterotrophic bacterial composition in the biomass was on an average 2% except for June-July 2014 (Fig. 1b). This increase in the summer months of the second year was similar to the increase observed in the first year, where members of Chlorellaceae (Order: Chlorellales; Class: Trebouxiophyceae; Phylum: Chlorophyta) were the dominant microalgal taxa^6^.

### Indigenous microalgal diversity in HRAP

**Figure 2:**
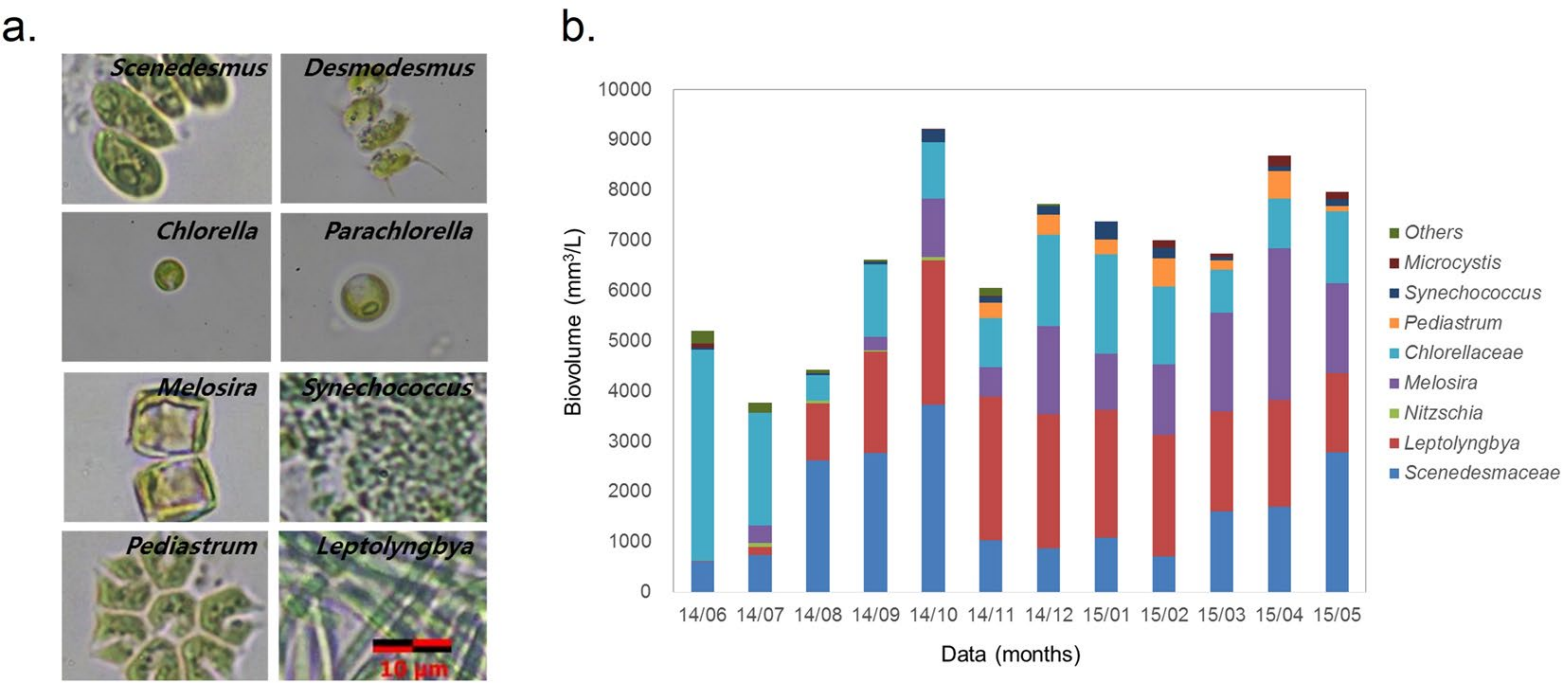
(**a**) Photomicrographs of dominant microalgal and cyanobacterial taxa found in HRAP. (**b**) Microalgal diversity in the HRAP based on biovolume calculations. Biovolume was calculated from cell numbers and volumetric data based on image analysis.

We attempted to calculate cell number and biovolume of distinct genera, or family in case of morphologically similar indigenous taxa in the HRAP. The photomicrographs of dominant microalgal species in HRAP are presented in Fig. 2a. Of these dominant strains, cyanobacterium *Leptolyngbya* dominated volumetrically, on an average 20% throughout the year. Unlike in the first year where *Synechococcus* sp. dominated numerically and because of its size could not dominate volumetrically, *Leptolyngbya* is a filamentous cyanobacterium and hence could dominate volumetrically^6^. Among the microalgae, taxa of the family Chlorellaceae (*Chlorella*, *Parachlorella* and *Dictyosphaerium*) and family Scenedesmaceae (*Scenedesmus* and *Desmodesmus*) dominated (Fig. 2b). *Pediastrum* was one of the few species, which was dominant, especially during winter months in spite of the temperature control^6, 11^. The presence of cyanobacteria was attributed to the cyanobacterial blooms present in influent wastewater streams, which contributes to the microbial diversity of the HRAP. The cyanobacterial population was more prevalent in the bloom and post bloom period (August 2014–January 2015), which subsequently decreased giving way to Chlorellaceae and Scenedesmaceae, which became dominant during spring and summer months (February 2015–May 2015; June 2014–July 2014, respectively). Although diatoms were dominant taxa in the second year, diatoms were not consistently present throughout the operating period (2013–2015). In order to determine how microalgal diversity was influenced by environmental, meteorological and biological factors, principal component analysis (PCA) was performed using data of microalgal biomass, bacterial biomass, diversity indices of microalgae, 4 physico-chemical parameters, 9 meteorological parameters, and 8 parameters of influent wastewater quality, which corresponded to a total of 36 components.

### Multivariate analysis of factors influencing microalgal diversity in HRAP

**Figure 3:**
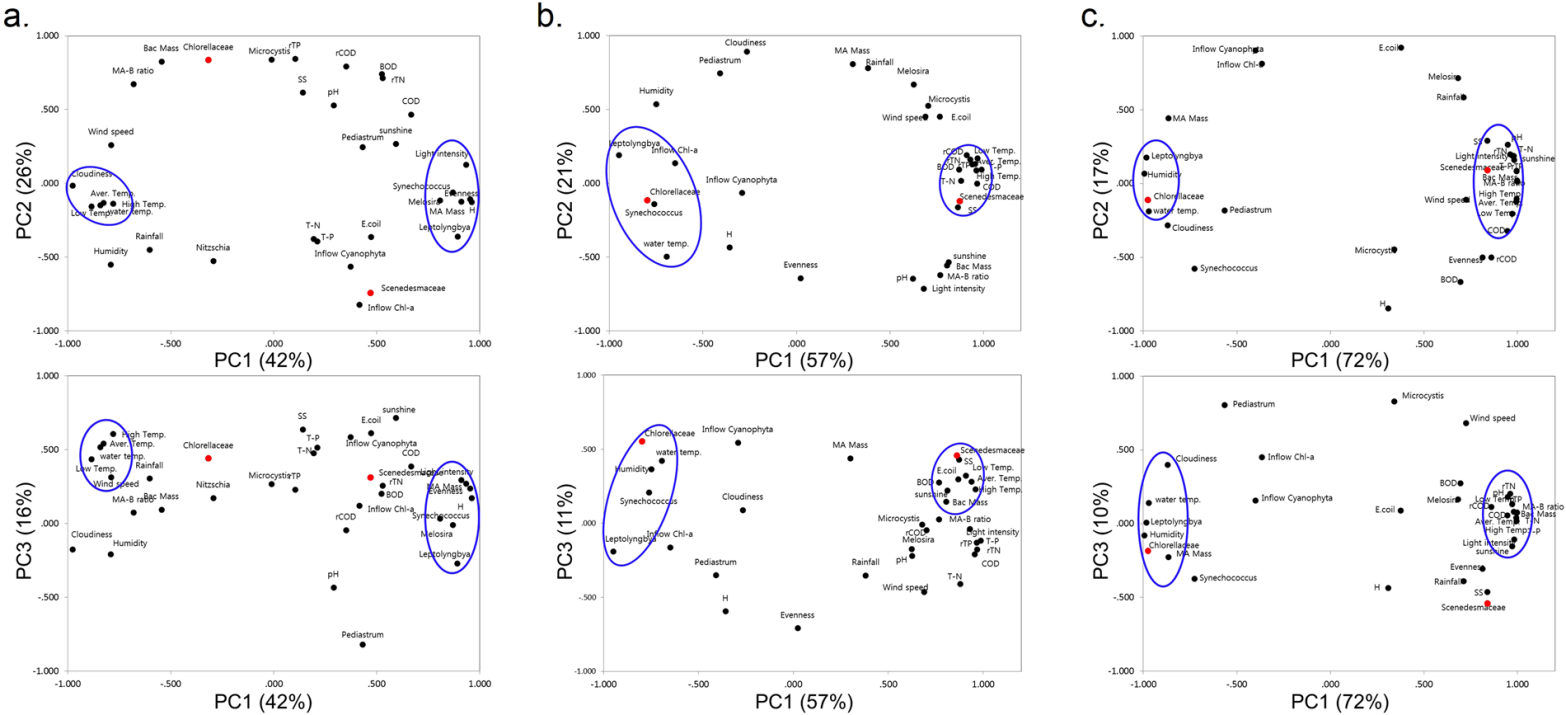
A 36-component 2D-PCA scatter plot based on correlation coefficients of all factors before (**a**), after (**b**) and during (**c**) temperature control experiments in HRAP. High correlation coefficient cluster is represented by bold blue line circle whereas dominant microalgal groups can be seen as red spots. The PCs were selected based on Scree plot and without rotation.

PCA was performed before the temperature control (June 2014–November 2014), during temperature control (December 2014–February 2015) and after temperature control (March 2015–May 2015). The effect of temperature was clearly reflected in the PC analysis, as measured water temperature was clustered with meteorological parameters – low, average and high air temperature – before temperature control (Fig. 3a). However, during and after temperature control, water temperature was distinctly clustered from air temperature (Fig. 3b and c). The PC analysis during and after temperature control, in winter and spring months, respectively, were similar (Fig. 3b and c). The variance of PC1, PC2 and PC3 before temperature control was 42%, 26% and 16%, while ‘during’ and ‘after’ temperature control experiments were 72%, 17% and 10%, and 57%, 21% and 11%, respectively (Fig. 3). Interestingly, PC analysis of ‘before’ temperature control data reveals that both species evenness index and Shannon’s diversity index (H) were clustered together with microalgal biomass. But Chlorellaceae and Scenedesmaceae were neither clustered together nor had high correlation coefficients with that of meteorological parameters or diversity indices (Fig. 3a). While, meteorological parameters like air temperature and cloudiness was negatively correlated with that of microalgal biomass and diversity, light intensity was clustered with microalgal biomass and diversity, and showed a positive correlation. Previous studies have noted that temporal weather changes are major impediments on phytoplankton abundance and diversity^13–15^. Our results prove that in atmospheric fluctuations (before temperature control), diversity plays an important part in maintaining microalgal biomass productivity (Fig. 3a, Table S2).

Subsequently, temperature control in winter months and thereafter resulted in disturbing the natural selection process in the HRAP. This was manifested in the PC analysis of datasets during and after temperature control. In ‘during’ and surprisingly, in ‘after’ temperature control experiments, water temperature was not clustered with air temperature and was negatively correlated. Importantly diversity indices were not clustered with microalgal biomass (MA Mass), neither positively correlated. In both these cases, increased water temperature favoured Chlorellaceae growth but meteorological parameters like air temperature, wastewater characteristics during this period encouraged Scenedesmaceae. This resulted in skewed species diversity and evenness, favouring Chlorellaceae growth (Figs 2 and 3b,c)^6, 13^. Meanwhile, Chlorellaceae population continued to flourish even after temperature control. But influent microbial diversity had an overarching role on biomass productivity as indicated by the growth of *Melosira* sp. and *Pediastrum* sp. in spite of temperature control (Fig. 2b).

Taken together, multivariate analyses clearly revealed that species diversity would aid biomass productivity in this engineered ecosystem, like terrestrial and aquatic ecosystems^1, 16, 17^. The temperature control event altered the natural selection process but maintained high biomass productivity. Nevertheless, there was also a possibility of this event increasing vulnerability for crashes due to meteorological conditions or predators. To test these possibilities, we isolated, identified and cultivated most cultivable, dominant species of microalgae present in HRAP. The optimum conditions and tolerance of each strain was tested to understand their role in HRAP and their relation to microalgal dynamics in open pond cultivation.

### Optimization of culture conditions of dominant, indigenous microalgae in HRAP

**Figure 4:**
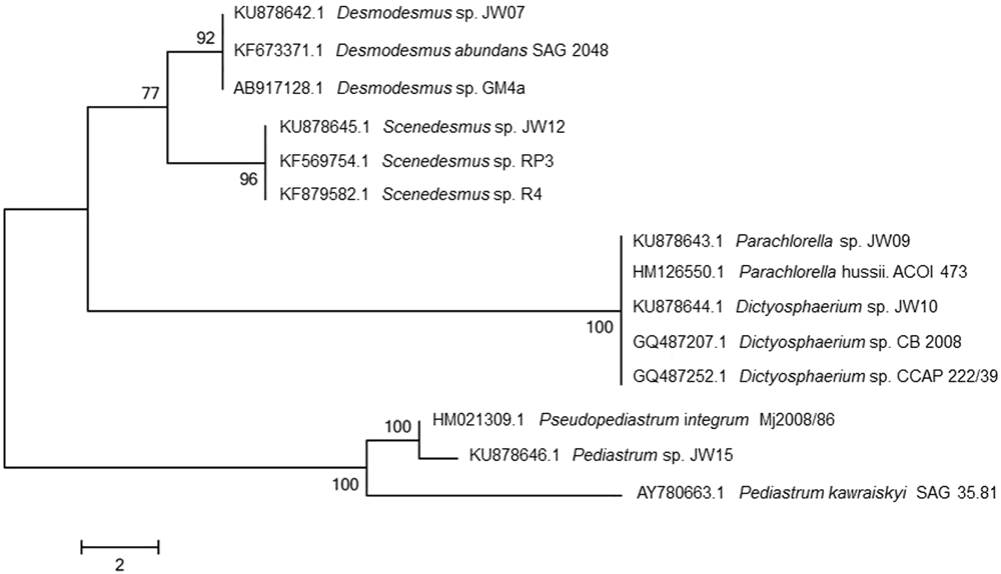
Phylogenetic tree of the isolated strains – JW07, JW09, JW10, JW12 and JW15 performed by neighbour-joining algorithm in the MEGA 5 software with bootstrap values based on 1000 replications.

**Figure 5:**
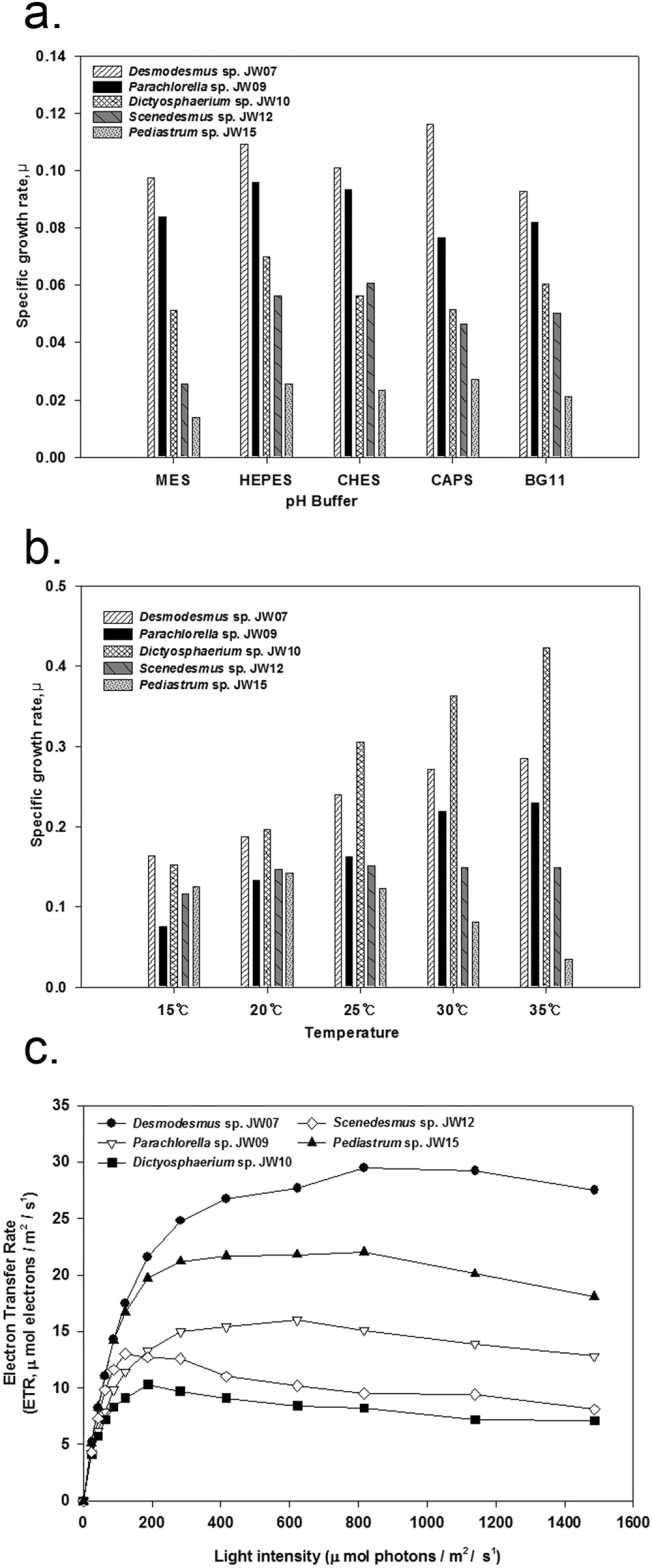
Specific growth rate and electron transfer rate of different microalgal strains under various pH and temperature profiles (**a,b**), and different light intensities (**c**), respectively.

All dominant, cultivable, unialgal strains from HRAP were isolated using micropicking followed by continuous serial dilutions^18^. Further morphological analysis followed by phylogenetic analysis of the unialgal strains revealed that these strains were *Pediastrum* sp., *Parachlorella* sp., *Dictyosphaerium* sp., *Desmodesmus* sp., and *Scenedesmus* sp. (Figs 2a and 4). Each of these strains was subjected to different pH (5.5–11.0), temperature (15–35 °C) and light intensity profiles (0–1600 µmol photons/m^2^/s). The temperature profile revealed that *Desmodesmus* sp., *Dictyosphaerium* sp., and *Parachlorella* sp. showed highest specific growth rates (µ) at 35 °C while *Scenedesmus* sp. and *Pediastrum* sp. showed highest µ at 25 °C and 20 °C, respectively. (Fig. 5a and b). On the contrary, although *Pediastrum* sp. was the least tolerant strain to changes in temperature with 75.85% reduction in µ, *Scenedesmus* sp. was the most resistant to temperature change with just 22% reduction in µ. However, *Scenedesmus* sp. had low µ (0.1518) compared to *Dictyosphaerium* sp. (0.4234), which was the highest among all strains. *Pediastrum* sp. had higher µ at lower temperatures (15–20 °C), compared to higher temperatures.

The pH profile experiment clearly revealed that *Scenedesmus* sp. and *Pediastrum* sp. had least tolerance to pH changes with reduction in µ of 57.82% and 48.33%, respectively. Other strains showed relatively higher resistance with an average of 22.31% reduction in µ. Similarly, the ETR of *Desmodesmus* sp. and *Pediastrum* sp. were relatively higher than other strains studied, but *Dictyosphaerium* sp. had low ETR at higher light intensities (Fig. 5c). *Dictyosphaerium* sp. and *Scenedesmus* sp. exhibited respective highest ETR at light intensity of ~150 μmol photons m^−2^ s^−1^ while *Desmodesmus* sp. and *Pediastrum* sp. exhibited respective highest ETR at light intensity of >500 μmol photons m^−2^ s^−1^. It must be noted that the average annual light intensity in the greenhouse hosting HRAP was 700 μmol/m^2^/s.

Therefore, different strains exhibited different pH, temperature and light intensity optimums as well as differential relative tolerance to these major environmental factors. Moreover, lab scale experiments helped validate outdoor cultivation results. For instance, *Pediastrum* sp. had high affinity to low temperatures in lab scale experiments, which corroborated with the occurrence of *Pediastrum* specifically during winter months in outdoor cultivation as a part of influent microbial consortium even though water temperature was controlled in HRAP^11, 12^. Similarly, Scenedesmaceae and Chlorellaceae dominated almost throughout the year, which reflects the tolerance of these strains as ascertained by lab scale experiments. Taken together, these results show that microalgal diversity helps in tolerating adverse environmental conditions, thereby maintaining a critical microalgal-bacterial ratio, allowing microalgal dominance in HRAP treating wastewater^19^. Moreover, wastewater inflows have highly fluctuating conditions such as differing nutrients, pH and organic carbon levels, therefore, different strains suited to different conditions need to be encouraged^15, 16^.

Further experiments showed that the total lipid content of the strains ranged from 26% to 40%, with *Dictyosphaerium* sp. possessing the highest lipid content of 39.57 ± 3.89%, followed by *Pediastrum* sp. with 39.16 ± 0.28% and *Desmodesmus* sp. showing lowest lipid content of 26.84 ± 1.26% under normal growth conditions (Table 1). The feedstocks rich in MUFA, especially C18 fatty acids; oleic acid (C18:1n9c), are suitable for biodiesel production^20^. All the strains had high mono unsaturated fatty acid (MUFA), poly unsaturated fatty acid (PUFA) ratio of >2, except for *Desmodesmus* sp. (1.19) and *Pediastrum* sp. (1.25) (Table S3). However, *Pediastrum* sp. had low saturated fatty acid (SFA) content leading to a relatively higher MUFA/SFA ratio of 1.42, comparable to *Parachlorella* sp. with MUFA/SFA ratio of 1.39. Incidentally, *Parachlorella* sp. had a high MUFA/PUFA ratio of 2.66 with 38.6% Oleic acid making the algae an ideal feedstock for biodiesel production. MUFA methyl esters, especially oleic acid, are considered to be better than PUFA methyl esters, because oil with high oleic acid content has been reported to be a reasonably balanced fuel. Its ignition quality, combustion heat, cold filter plugging point, oxidative stability, viscosity and lubricity, which are determined by the structure of its component fatty esters, are better suited for biodiesel applications^21, 22^. Therefore, the resulting biomass after wastewater treatment could be used as a biodiesel feedstock (Table S3).

**Table 1:** Biomass density and lipid content of the isolated microalgal strains.

Microalgal strain	Biomass density	Maximum lipid content	
	(g L^−1^)	(g L^−1^)	(%)
*Desmodesmus sp*. JW07	1.48 ± 0.53	0.39 ± 0.18	26.84 ± 1.26
*Parachlorella sp*. JW09	1.89 ± 0.14	0.64 ± 0.12	33.63 ± 3.71
*Dictyosphaerium sp*. JW10	2.76 ± 0.02	1.09 ± 0.11	39.57 ± 3.89
*Scenedesmus sp*. JW12	2.02 ± 0.11	0.66 ± 0.05	33.44 ± 0.38
*Pediastrum sp*. JW15	1.50 ± 0.39	0.59 ± 0.15	39.16 ± 0.28

### Predatory pressure in HRAP

**Figure 6:**
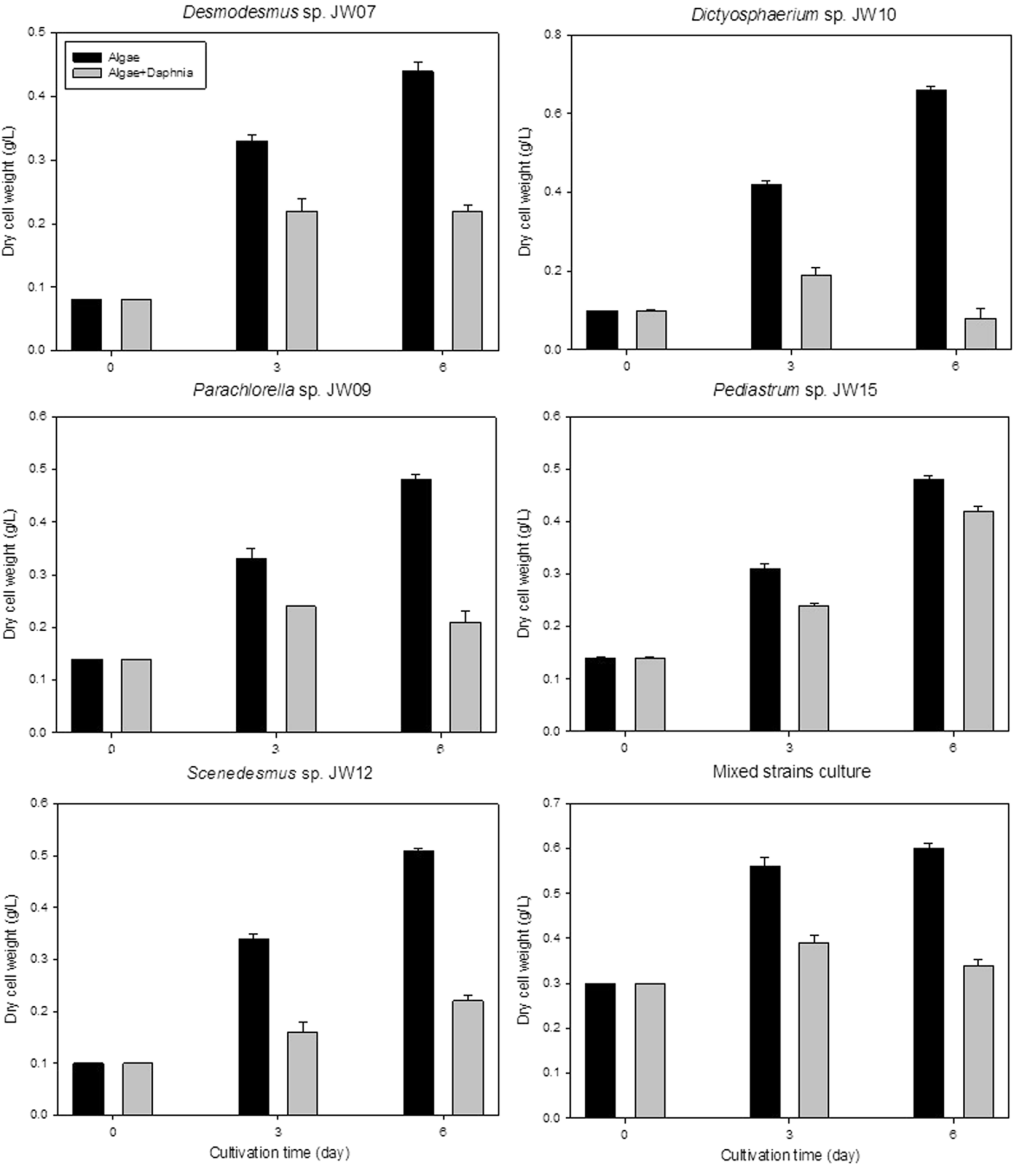
Co-cultivation study of isolated, indigenous microalgal strains with *Daphnia* isolated from the HRAP.

Another challenge in outdoor microalgal cultivation in HRAP is the predatory pressure from zooplankton, which are often difficult to avoid in wastewater treatment systems^23^. Cladoceran *Daphnia* sp. is a prevalent genus reported in most microalgae based wastewater treatment systems and *Daphnia* sp. was also predominant in our HRAP. The indigenous species was isolated and identified based on morphological analysis (Fig. S1)^24^. *Daphnia* was then co-cultivated with each of the associated microalgae and effect of zooplankton on microalgal growth was tested. Effect of *Daphnia* on microalgal consortium (all five strains co-cultivated together) was also tested to mimic open pond cultivation in lab scale. Among the microalgal strains, *Dictyosphaerium* sp. was the most palatable strain for *Daphnia* sp. with 87.87% reduction in dry cell weight (DCW) on the 6^th^ say of cultivation followed by *Scenedesmus* sp. (56.86%) while *Pediastrum* sp. (12.5%) and mixed strains culture (43.3%) were most resistant to *Daphnia* predation (Fig. 6). *Pediastrum* sp. is a large sized microalga which makes it difficult to ingest for *Daphnia*. Both *Desmodesmus* sp. and *Parachlorella* sp. (50%) also nourished *Daphnia* growth as the cultures with *Daphnia* showed just half the cell numbers compared to the control. All cultures showed reduced DCW or unsubstantial increase in DCW with cultivation time but *Pediastrum* sp. showed 42.85% increased DCW with cultivation time. Interestingly, mixed culture was more resistant to *Daphnia* compared to individual strains except *Pediastrum* sp., showing that microalgal diversity would yield better productivity in outdoor cultivation because of differential herbivore resistance as observed in other natural terrestrial and aquatic ecosystems^1, 17, 25^.

It must be noted that *Dictyosphaerium* sp. possessed the highest biomass density and lipid content (Table 1). Moreover, this strain also had high temperature and pH tolerance. However, *Dictyosphaerium* sp. was the least tolerant strain for *Daphnia* forage. In fact, after 6 days of growth, the *Daphnia* co-cultivated culture had about 10% cell numbers to that of control culture. These results show that this strain was vulnerable in outdoor cultivation and selective cultivation of this strain, which had the highest biomass density among the isolated Chlorellaceae strains, would probably lead to crashes. Similarly, *Parachlorella* sp. among Chlorellaceae strains had high tolerance to different temperature and pH profiles, but performed poorly to herbivore predation. Thus Chlorellaceae, which was selectively enriched because of the temperature control in winter months and thereafter as seen earlier, would not survive very well under predatory pressure. In contrast, *Desmodesmus* sp. which had high pH, light and temperature tolerance also had relatively good resistance to *Daphnia*. However, Scenedesmaceae which also includes *Desmodesmus* sp. was not selectively enriched following temperature control, in spite of being favoured under the influent wastewater and meteorological conditions (Figs 3 and 5). Likewise, *Pediastrum* sp. was favoured at low temperature in the winter months^11, 12^, but temperature control allowed Chlorellaceae to dominate leaving the HRAP vulnerable to possible predation and environmental fluctuations (Figs 2 and 6). However, *Pediastrum* sp. was still able to grow which highlights that influent microbial diversity and other environmental factors would still influence microalgal growth in this engineered ecosystem. Therefore, an induced temperature control change would transition the innate microalgal diversity of the ecosystem which was favoured by these influencing factors. This leads to skewed species evenness, and enriching certain species might be detrimental to the whole ecosystem, especially in fluctuating wastewater treatment processes^26^.

These results prove that diversity, niche or seasonal partitioning and mutualistic growth depending on biotic and abiotic factors are important. The relative dominance of one species over the other and inter-species adaptation based on niche and predation is deprived in mono-cultivations negatively affecting productivity. These results would be the first starting point for simulating ecosystem productivity in engineered ecosystems particularly open ponds with limited trophic levels. It must be noted that presence of higher trophic levels would drastically alter herbivory and therefore might positively affect ecosystem productivity^25^. The temperature control study and resultant PC analyses clearly indicated the effect of temperature on biomass productivity and the indoor lab scale experiments suggested a possible change in diversity with change in temperature. Earlier studies have shown a combination of abiotic factors like temperature and other biotic factors like herbivory control microalgal diversity and productivity^25, 26^. Our results also suggest that effect of abiotic factors like temperature get strengthened in combination with strong herbivory presence. Ultimately the combined effect is the loss of diversity and progression towards monocultures and ecosystem vulnerability^8, 16, 17^.

In précis, the study confirms that different indigenous strains within HRAP had different tolerance levels to pH, light, temperature and predatory pressure. These factors adversely affect the functioning of this engineered ecosystem, creating various ‘checks’ on biomass productivity. But high microalgal diversity counterbalances these factors to ensure stable biomass productivity resulting in system fluidity amid fluctuations. On the other hand, selective enrichment supports system rigidity and therefore vulnerability to crashes. The study provides the first comprehensive analysis of factors influencing ecosystem productivity in ever-increasing algal open cultivation systems for environmental and biotechnological applications. Although performed on engineered ecosystem, the results of this multi-dimensional study carries significance for natural ecosystems, and the effect of climate phenomenon like global warming on natural ecosystems.

## Methods

### HRAP operation and wastewater treatment

The water samples for microalgae isolation were collected in triplicates from small scale HRAP operated for 2 years (May 2013–May 2015), using sewage from Daejeon sewage treatment plant. Details of influent characteristics, biomass yield and meteorological parameters are given in Supplementary Information (Table S1). Other wastewater influent characteristics were obtained from the respective agency (http://water.nier.go.kr). Sunlight was the only light source. The average light intensity, in terms of average peak daylight, for two years was 700 μmol/m^2^/s with natural light/dark cycles. The temperature was controlled at 20 °C in winter months (December 2014–February 2015) in the greenhouse where the small scale HRAPs was stationed. Other months, ambient air temperature was maintained. As the experiment was done at a larger scale, a control set with ambient temperature during the temperature control experiment would not be established. But the previous study (May 2013–May 2014) would serve as a valid reference point as it was carried out completely under prevailing ambient conditions^6^. The HRAP design and operational parameters were as described in the previous study^6^.

### Chemical analyses, meteorological data and statistical analyses

All chemical and biological analyses were performed as mentioned in the previous study^6^. Briefly, all samples were filtered through 0.2 μm membranes (Minisart, Sartorius Stedim Biotech SA, Germany) before analysis. The concentrations of TN and NH_3_–N, and TP were determined using second-derivative method and ascorbic acid method, respectively, as prescribed in standard methods^27^. Culture pH and water-temperature were measured using an electrode (pH 3110 SET 2/SenTix® 41, WTW, Germany). The COD was measured according to standard methods^27^. Meteorological data was obtained from Korea Meteorological Agency (http://www.kma.go.kr). The data thus obtained was Z-transformed so that the mean and variance corresponded to 0 and 1^28^. Principal component analysis (PCA) and Pearson’s linear correlation analysis were performed as mentioned before^6, 28^ (Table S2). All statistical analyses were computed using SPSS 18 (SPSS Inc., USA).

### Microalgal diversity analysis

For microalgal diversity analysis, morphological and genetic analysis of each microalga was performed as mentioned before^6^. Briefly, all cultures were grown in BG11 agar plates and liquid medium in triplicates with a continuous illumination at a light intensity of 130 µmol·m^−2^·s^−1^ and temperature of 25 °C for seven days. Isolated prokaryotic and eukaryotic strains were identified by sequencing 16S rRNA gene and 18S rRNA gene, respectively^18^. The details of the primer sets are described in the succeeding section. Isolated diatom strain was identified by diatom identification primer (D512F, D978R)^29^. The diversity indices were also determined as previously reported^28^. For biovolume calculations, each dominant taxa or group was identified and computed using ImageJ software as described in the earlier study^30^. In case of Chlorellaceae, various genera like *Chlorella*, *Parachlorella* and *Dictyosphaerium* dominated, in the second year of operation (June 2014–May 2015). But these taxa were not easily distinguishable using the above method as microalgae flocs were rapid and prevalent in HRAP^31^. Therefore, these genera were grouped together as Chlorellaceae. Similarly in the case of Scenedesmaceae, genera *Scenedesmus* and *Desmodesmus* were grouped together.

### Isolation and identification of dominant microalgae from HRAP

Five microalgae strains were isolated by using of a micropipette under a microscope (Nikon F, Japan) followed by dilution in liquid and solid BG11 medium^18^. Choice of microalgae was made based on dominance through biovolume calculations, and cultivability. *Melosira* sp. was the only notable species although abundant based on biovolume in the outdoor cultivation, could not be cultivated in the laboratory. *Microcystis* and *Leptolyngbya* were not cultivated as some species of the genera are noxious and undesirable for commercial production. Finally, five unialgal strains were purified by repeated subculture and confirmed for purity by periodic monitoring using a light microscope (Nikon F, Japan) connected to a camera and computer.

The cyanobacteria and microalgal strains were identified morphologically on the basis of microscopic observation and further confirmed by 16S and 18S rRNA sequencing, respectively. Genomic DNA was isolated from the microalgal biomass by G-spin plant DNA extraction kit (Intron, Seoul, South Korea) according to the manufacturer’s instructions. Two kinds of primers, 27F (5′-AGAGTTTGATCCTGGCTCGA-3′) with 518R (5′-ATTACCGCGGCTGCTGG-3′) and 165F (5′-CGACTTCTGGAAGGGACGTA-3′) with 1780R (5′-CTAGGTGGGAGGGTTTAATG-3′) were used for PCR amplification of 18S rRNA genes. For 16S rRNA analysis, 27F (AGAGTTTGATCMTGGCTCAG) and 1492R (CGGTTACCTTGTTACGACTT) primers were used. Each 50 μL PCR mixture contained 100 nmol of primers, 100 μM dNTPs, 5 mL of 10× PCR buffer, 0.25 mL of 5 U *EXTaq* polymerase (TaKaRa, Tokyo, Japan), 2.5 μL of 2% bovine serum albumin and DNA. Samples were amplified as following: 95 °C for 5 min, 30 cycles of denaturation (1 min at 94 °C), annealing (1 min at 60 °C), and extension (1 min at 72 °C), and a final extension at 72 °C for 10 min. Amplified PCR product was examined by 1% agarose gel electrophoresis. The PCR products were purified using a gel extraction kit (Solgent, Seoul, Korea) and were sequenced with an ABI Prism 377 automated sequencer (Applied Biosystems, CA, USA). Sequences were then compared with those in the GenBank database using the BLASTN facility of the National Center for Biotechnology Information (NCBI, http://ncbi.nlm.nih.gov/) and deposited under accession numbers *Desmodesmus* sp. (KU878642), *Scenedesmus* sp. (KU878645), *Parachlorella* sp. (KU878643)*, Dictyosphaerium* sp. (KU878644) and *Pediastrum* sp. (KU878646). This was followed by sequence alignment and phylogenetic analysis^18, 32–34^.

### Physiological analysis of isolated strains

#### Effect of Temperature

Prior to lab-scale based experiments, all strains were initially screened with PhotoBiobox^TM^ (Shinhwa Science, Korea) for optimal conditions (data not shown)^35^. Subsequently, the influence of temperature on the isolate was assessed under different temperatures – 15 °C, 20 °C, 25 °C, 30 °C and 35 °C. The isolates were cultured in modified BG11 with increased P source (4X) and incubated at above mentioned temperatures. Biomass productivity was analyzed over a period of 28 days. All the experiments were performed in triplicates in 250 ml flask with 50 ml working volume, in shaking incubator at 130 rpm and with a continuous illumination at a light intensity of 130 µmol·m^−2^·s^−1^, until otherwise mentioned.

### Effect of Light Intensity

Photosynthetic activity of isolated microalgae was estimated using JUNIOR-PAM Chlorophyll Fluorometer (Heinz Walz GmbH, Germany) with its operational software, WIN-CONTROL-3 (Heinz Walz GmbH, Germany)^36^. The photosynthesis Yield Analyzer MINI PAM II has been designed for highly sensitive saturation pulse analysis of photosystem II. Reliable PAR and Y(II) data form the basis for calculations of electron transport rates (ETR) according to following equation.

$${\rm{ETR}}={\rm{PAR}}\cdot \mathrm{ETR} \mbox{-} \mathrm{factor}\cdot {{\rm{P}}}_{{\rm{PS}}2}/{{\rm{P}}}_{{\rm{PPS}}}\cdot {\rm{Y}}({\rm{II}})$$

ETR is obtained by multiplying, Y(II), the effective photochemical quantum yield of PS II, by an estimate for the photon flux density absorbed by PS II. The ETR-factor corresponds to the ratio of photons absorbed by photosynthetic pigments to incident photons. In this study, the value for ETR-factor was 0.84 which matches reasonably well with average absorbance in the visible range of green algae. And P_PS2_/P_PPS_ value of 0.5 is a constant based on the assumption that both photosystems Ι and II absorb equal amount of photon energy during the measurement. The light efficiency yield curve plotting ETR versus PAR, revealed the effect of light intensity on isolated microalgae.

### Effect of pH

To evaluate the effect of pH on isolates, they were cultured in modified BG-11 media adjusted to pH 5.5, 7, 9 and 11 using biological buffers; MES(2-(N-morpholino) ethanesulfonic acid), HEPES(4-(2- hydroxyethyl)piperazin-1-yl]ethanesulfonic acid, CHES((N-cyclohexyl-2-aminoethanesulfonic acid) and CAPS(N-cyclohexyl-3-aminopropanesulfonic acid), respectively. Modified BG-11 medium (initial pH 6.5) was taken as control in this experiment. The biomass productivity and specific growth rate were calculated based on optical density measurements (680 nm) using spectrophotometer after calibrations with standards (Sunrise, Tecan, Austria) and the pH was analyzed by pH meter (LAQUAtwin, HORIBA, Japan)^32^.

### Effect of Zooplankton

Among the few zooplankton species found in the HRAP, *Daphnia* sp. was the most prevalent and dominant. *Daphnia* sp. was identified based on morphological analysis in an optical microscope (Nikon F, Japan) and comparing the same with widely used taxonomic keys^24^. For co-cultivation, 20 individuals of *Daphnia* were selectively inoculated with each alga under optimal conditions for the respective strain as obtained from physiological analyses and the growth of microalgae was monitored by dry cell weight as mentioned before^32^. All mixed culture experiments except for physiological analyses were performed in triplicates in 250 ml flask with 50 ml working volume, in shaking incubator at 130 rpm and with a continuous illumination at a light intensity of 130 µmol·m^−2^·s^−1^ with a starting pH of 6.5 and temperature of 25 °C.

### Lipid analyses

The total lipids were extracted by mixing chloroform-methanol solvent mixture using a slightly modified version of Bligh and Dyer’s method^37–39^. FAME analysis was performed using gas chromatography fitted with flame ionization detector and the rest of the protocol was as mentioned elsewhere^40^.

## Electronic supplementary material

Table S1-S3, Figure S1
