# Supplementary material for: Microalgal diversity fosters stable biomass productivity in open ponds treating wastewater
Source: Sci Rep. 2017 May 16;7:1979. doi: 10.1038/s41598-017-02139-8 (PMC5434013; doi:10.1038/s41598-017-02139-8)
Supplement: Supplementary file 1 — Table S1-S3, Figure S1 [file 41598_2017_2139_MOESM1_ESM.doc]

**Supplementary Information**

**Microalgal diversity fosters stable biomass productivity in open ponds treating wastewater**

Dae-Hyun Cho^1#^, Jung-Woon Choi^1,2#^, Zion Kang^1,3^, Byung-Hyuk Kim^1^, Hee-Mock Oh^1,2^, Hee-sik Kim^1,2*^, Rishiram Ramanan^4*^

^1^Cell Factory Research Center, Korea Research Institute of Bioscience and Biotechnology (KRIBB), Yuseong-gu, Daejeon 305-806, Republic of Korea

^2^Green Chemistry and Environmental Biotechnology, University of Science and Technology (UST), Yuseong-gu, Daejeon 305-350, Republic of Korea

^3^Department of Chemical and Biomolecular Engineering, Korea Advanced Institute of Science and Technology (KAIST), Daejeon 305-701, Republic of Korea

^4^Department of Environmental Science, School of Earth Science Systems, Central University of Kerala, Kasaragod District, Kerala, India

*Corresponding authors:

Dr. Hee-Sik Kim

Cell Factory Research Center, KRIBB, Daejeon 305-806, Republic of Korea

Tel. +82-42-860-4326; Fax. +82-42-879-4594

E-mail: [hkim@kribb.re.kr](mailto:hkim@kribb.re.kr)

Dr. Rishiram Ramanan

Department of Environmental Science, School of Earth Science Systems, Central University of Kerala, Kasaragod District, Kerala, India

Tel. +91-467-2285494; Fax. +91-467-2232402

E-mail: [rishi@cukerala.ac.in](mailto:rishi@cukerala.ac.in)

^#^These authors contributed equally

**Table S1.** Yearly data (June 2014-May 2015) summary of microalgal composition and diversity, physicochemical, meteorological and influent wastewater parameters

| Date | Year-Month | 14-6 | 14-7 | 14-8 | 14-9 | 14-10 | 14-11 | 14-12 | 15-1 | 15-2 | 15-3 | 15-4 | 15-5 |
| --- | --- | --- | --- | --- | --- | --- | --- | --- | --- | --- | --- | --- | --- |
| Microalgal  composition  (g/L) | *Scenedesmaceae* | 0.0583758 | 0.072474 | 0.255436 | 0.269412 | 0.363786 | 0.100876 | 0.084302 | 0.105957 | 0.069557 | 0.155833 | 0.164924 | 0.270869 |
|  | *Leptolyngbya* | 0.0019639 | 0.014857 | 0.110449 | 0.197379 | 0.279513 | 0.278231 | 0.260996 | 0.246467 | 0.235085 | 0.194129 | 0.207182 | 0.153383 |
|  | *Nitzschia* | 0 | 0.007649 | 0.005544 | 0.001714 | 0.006353 | 0 | 0 | 0 | 0 | 0 | 0 | 0 |
|  | *Melosira* | 0 | 0.033518 | 0 | 0.026562 | 0.113724 | 0.056925 | 0.170184 | 0.109347 | 0.136545 | 0.19244 | 0.294549 | 0.175373 |
|  | *Chlorellaceae* | 0.4095559 | 0.219475 | 0.049643 | 0.140653 | 0.109412 | 0.095743 | 0.17768 | 0.19316 | 0.151336 | 0.083134 | 0.096962 | 0.138361 |
|  | *Pediastrum* | 0 | 0 | 0 | 0 | 0 | 0.02952 | 0.038709 | 0.028279 | 0.054995 | 0.017566 | 0.052762 | 0.010439 |
|  | *Synechococcus* | 0.0045442 | 0 | 0.004197 | 0.005193 | 0.023966 | 0.012348 | 0.017018 | 0.035651 | 0.020827 | 0.007011 | 0.009539 | 0.012941 |
|  | *Microcystis* | 0.0080098 | 0 | 0 | 0 | 0.002152 | 0.001238 | 0 | 0 | 0.013649 | 0.005962 | 0.020759 | 0.015342 |
|  | Others | 0.0247961 | 0.020007 | 0.005996 | 0.003264 | 0 | 0.015245 | 0.003471 | 0 | 0 | 0 | 0 | 0 |
| HRAP  data | Algal Mass (g/L) | 0.5072456 | 0.36798 | 0.431266 | 0.644177 | 0.898905 | 0.590126 | 0.752361 | 0.718861 | 0.681993 | 0.656075 | 0.846677 | 0.776708 |
|  | Bacterial Mass (g/L) | 0.0327544 | 0.02402 | 0.008735 | 0.005823 | 0.005095 | 0.012374 | 0.003639 | 0.003639 | 0.008007 | 0.018925 | 0.005823 | 0.023292 |
|  | Bacterial/algae (%) | 6.065625 | 6.127519 | 1.985114 | 0.895846 | 0.56362 | 2.053755 | 0.481399 | 0.50372 | 1.16038 | 2.803667 | 0.68305 | 2.9115 |
|  | pH (ppm) | 9.26 | 8.792 | 9.065 | 8.7075 | 9.164 | 9.2525 | 9.034 | 8.8725 | 9.085 | 9.4575 | 8.945 | 9.278 |
|  | COD (ppm) | 89.675 | 85.28 | 67.575 | 70.65 | 88.74 | 88.175 | 73.34 | 81.175 | 85.65 | 91.05 | 98.2 | 99.24 |
| Meteorological  data | Light intensity (µE) | 794.75 | 759.2 | 655.75 | 887.75 | 975.2 | 881 | 853.4 | 843.5 | 864.75 | 1108 | 825.25 | 1066.2 |
|  | Water temp. (℃) | 24.9675 | 28.058 | 25.725 | 23.5425 | 17.398 | 10.5675 | 23.8 | 24.05 | 23.825 | 18.575 | 14.595 | 21.672 |
|  | Aver. temp. (℃) | 22.9 | 25.9 | 24.2 | 21.6 | 14.9 | 8.5 | -1.3 | 0 | 1.9 | 7.2 | 13.3 | 19.5 |
|  | Low temp. (℃) | 18.9 | 22.5 | 21.4 | 17.1 | 9.5 | 4 | -5.5 | -4.4 | -2.5 | 0.6 | 7.8 | 13 |
|  | High temp. (℃) | 27.7 | 30.2 | 27.8 | 26.9 | 21.2 | 13.9 | 3.4 | 5 | 7.2 | 14.3 | 19.3 | 26 |
|  | Rainfall (mm) | 143.7 | 177.2 | 240.9 | 118 | 169.4 | 40.7 | 36.7 | 31.5 | 27 | 44.7 | 95.2 | 28.9 |
|  | Wind speed (m/s) | 1.7 | 1.6 | 1.6 | 1.3 | 1.4 | 1.2 | 1.4 | 1.3 | 1.7 | 1.7 | 1.9 | 1.6 |
|  | Humidity (%) | 77.1 | 82.6 | 87 | 78.2 | 76.3 | 76.5 | 74.7 | 72.7 | 66.2 | 51 | 68.1 | 59.8 |
|  | Sunshine (hr) | 6.7 | 6.1 | 4.2 | 6.9 | 7.9 | 5.5 | 5.7 | 5.5 | 5.6 | 8.8 | 6.0 | 9.9 |
|  | Cloudiness (10%) | 6.6 | 7.2 | 7.9 | 5.1 | 3.4 | 4.9 | 4.5 | 4.5 | 5 | 3 | 5.6 | 4 |
| Information of  influent | Inflow Chl-a | 2.8 | 6.9 | 10.7 | 9.6 | 13.7 | 6.7 | 4.7 | 3.3 | 3.9 | 3.6 | 3.4 | 2.8 |
|  | Inflow Cyanophyta | 166 | 789 | 575 | 2,752 | 1,458 | 364 | 343 | 7 | 50 | 49 | 0 | 133 |
|  | COD (ppm) | 180 | 147.7 | 146.8 | 158.5 | 169.5 | 166.6 | 142.6 | 148.5 | 153 | 153.2 | 161.2 | 171 |
|  | SS (ppm) | 151.4 | 132.4 | 130.9 | 136.4 | 143 | 130.5 | 122.6 | 122.9 | 118.5 | 132.5 | 131.7 | 150 |
|  | TN (ppm) | 38.587 | 36.347 | 37.665 | 67.439 | 41.862 | 37.584 | 38.491 | 37.296 | 39.335 | 45 | 43.32 | 42 |
|  | TP (ppm) | 4.042 | 3.74 | 4.072 | 9 | 4.217 | 4 | 3.456 | 3 | 3.656 | 4 | 4.391 | 4.395 |
|  | N:P ratio | 9.5 | 9.7 | 9.2 | 7.5 | 9.9 | 9.4 | 11.1 | 12.4 | 10.8 | 11.3 | 9.9 | 9.6 |
|  | *E.coli* No. (No./mL) | 183,256 | 195,078 | 188,953 | 206,819 | 253,731 | 168,636 | 125,351 | 106,834 | 110,933 | 123,484 | 141,033 | 137,528 |
| Diversity indices | H | 0.8302419 | 0.300727 | 0.536446 | 1.451847 | 1.917204 | 1.256962 | 1.138734 | 1.578918 | 1.395516 | 1.459792 | 1.215605 | 1.135603 |
|  | Evenness | 0.399262 | 0.144619 | 0.299396 | 0.698191 | 0.92198 | 0.645951 | 0.547615 | 0.718597 | 0.671102 | 0.814725 | 0.584583 | 0.583585 |

**Table S2. Correlation coefficient values at three different stages of temperature control experiment for all parameters used in principal component (PC) analyses. Grey regions indicate meteorological factors correlated with diversity indices.**

| **Parameters** | **Component** | | | | **Parameters** | **Component** | | | **Parameters** | **Component** | | |
| --- | --- | --- | --- | --- | --- | --- | --- | --- | --- | --- | --- | --- |
|  | PC1(42%) | PC2(26%) | | PC3(16%) |  | PC1(57%) | PC2(21%) | PC3(11%) |  | PC1(72%) | PC2(17%) | PC3(10%) |
| Before temperature control (June 2014 – November 2014) | | | | | During temperature control (December 2014 – February 2015) | | | | After temperature control (March 2015 – May 2015) | | | |
| Cloudiness | -.977 | | -.015 | -.176 | T-P | .987 | .095 | -.117 | MA-B ratio | .997 | .013 | .075 |
| Evenness | .960 | | -.127 | .173 | rTP | .967 | .172 | -.175 | Bac Mass | .997 | .022 | .076 |
| H | .953 | | -.107 | .238 | COD | .964 | -.002 | -.131 | High Temp. | .995 | -.103 | .019 |
| Light intensity | .933 | | .128 | .269 | High Temp. | .959 | .088 | .229 | Humidity | -.995 | .068 | -.080 |
| MA Mass | .909 | | -.122 | .296 | rTN | .954 | .131 | -.209 | rTP | .994 | .084 | .075 |
| *Leptolyngbya* | .892 | | -.360 | -.272 | *Leptolyngbya* | -.949 | .191 | -.191 | T-P | .993 | .086 | .077 |
| Low Temp. | -.887 | | -.155 | .436 | Aver. Temp. | .938 | .131 | .280 | Aver. Temp. | .992 | -.122 | .041 |
| *Synechocystis* | .869 | | -.062 | -.009 | rCOD | .928 | .162 | -.038 | *Leptolyngbya* | -.984 | .178 | .007 |
| Aver. Temp. | -.843 | | -.148 | .517 | Low Temp. | .908 | .191 | .323 | Light intensity | .981 | .160 | -.107 |
| Water temp. | -.829 | | -.132 | .543 | T-N | .879 | .018 | -.410 | T-N | .979 | .189 | .082 |
| *Melosira* | .806 | | -.115 | .035 | *Scenedesmus* | .871 | -.120 | .430 | *Chlorella* | -.977 | -.112 | -.182 |
| Wind speed | -.791 | | .261 | .314 | SS | .862 | -.161 | .458 | Sunshine | .970 | .189 | -.152 |
| High Temp. | -.781 | | -.137 | .608 | Sunshine | .811 | -.533 | .222 | Low Temp. | .970 | -.205 | .132 |
| MA-B ratio | -.683 | | .674 | .074 | Bac Mass | .804 | -.557 | .146 | rTN | .959 | .199 | .204 |
| COD | .667 | | .467 | .389 | *Chlorella* | -.798 | -.111 | .555 | pH | .947 | .265 | .183 |
| Rainfall | -.604 | | -.449 | .305 | MA-B ratio | .766 | -.621 | .028 | COD | .945 | -.321 | .058 |
| rTP | .103 | | .846 | .231 | *E. coli* | .766 | .453 | .275 | Cloudiness | -.872 | -.282 | .400 |
| Microcystis | -.012 | | .839 | .268 | *Synechocystis* | -.761 | -.141 | .208 | MA Mass | -.867 | .444 | -.225 |
| *Chlorella* | -.319 | | .836 | .441 | Humidity | -.749 | .538 | .366 | rCOD | .859 | -.499 | .113 |
| Bac Mass | -.546 | | .827 | .095 | *Microcystis* | .702 | .526 | -.046 | *Scenedesmus* | .837 | .094 | -.539 |
| Inflow Chl-a | .415 | | -.821 | .120 | Water temp. | -.695 | -.496 | .423 | SS | .837 | .291 | -.463 |
| rCOD | .350 | | .795 | -.044 | Wind speed | .688 | .452 | -.463 | Evenness | .812 | -.499 | -.303 |
| *Scenedesmus* | .469 | | -.739 | .314 | Cloudiness | -.267 | .894 | .090 | Wind speed | .723 | -.110 | .682 |
| rTN | .528 | | .713 | .258 | MA Mass | .300 | .810 | .439 | Rainfall | .711 | .586 | -.388 |
| *Nitzschia* | -.294 | | -.525 | .173 | *Pediastrum* | -.409 | .746 | -.350 | *Melosira* | .680 | .714 | .165 |
| *Pediastrum* | .431 | | .248 | -.819 | Light intensity | .678 | -.712 | -.009 | *Pediastrum* | -.566 | -.183 | .804 |
| Sunshine | .593 | | .270 | .716 | *Melosira* | .625 | .671 | -.219 | Inflow Cyanophyta | -.402 | .902 | .157 |
| SS | .140 | | .615 | .638 | pH | .622 | -.645 | -.173 | *E. coli* | .375 | .922 | .091 |
| *E. coli* | .472 | | -.364 | .613 | Evenness | .021 | -.642 | -.708 | Inflow Chl-a | -.367 | .813 | .452 |
| Inflow Cyanophyta | .372 | | -.565 | .587 | H | -.359 | -.435 | -.594 | *Microcystis* | .339 | -.445 | .829 |
| T-P | .211 | | -.392 | .516 | Inflow Cyanophyta | -.293 | -.064 | .545 | H | .308 | -.846 | -.436 |
| T-N | .193 | | -.375 | .478 |  |  |  |  |  |  |  |  |

**Table S3. Fatty acid composition of the different microalgae isolated from HRAP**

| **Fatty acid** | **Fatty acid composition (wt %)** | | | | |
| --- | --- | --- | --- | --- | --- |
|  | ***Desmodesmus* sp. JW07** | ***Parachlorella* sp. JW09** | ***Dictyosphaerium* sp. JW10** | ***Scenedesmus* sp. JW12** | ***Pediastrum* sp. JW15** |
| Myristic acid (C14:0) | 2.2 | 0.7 | 5.2 | 4.5 | 2.9 |
| Palmitic acid (C16:0) | 15.1 | 30.2 | 30.9 | 16.1 | 11.6 |
| Palmitoleic acid (C16:1) | 9.1 | 6.3 | 6.9 | 9.2 | 6.6 |
| Strearic acid (C18:0) | 12.3 | 1.4 | 2.8 | 15.7 | 10 |
| Oleic acid (C18:1n9c) | 24.1 | 38.6 | 33.8 | 24.6 | 28.1 |
| Linoleic acid (C18:2n6c) | 22.6 | 15.3 | 13.2 | 15.1 | 23.4 |
| Linolenic acid (C18:3n3) | 5.4 | 1.6 | 1.9 | 0.7 | 4.3 |
| Others | 9.2 | 5.9 | 5.3 | 14.1 | 13.1 |
| MUFA | 33.2 | 44.9 | 40.7 | 33.8 | 34.7 |
| PUFA | 28 | 16.9 | 15.1 | 15.8 | 27.7 |
| SFA | 29.6 | 32.3 | 38.9 | 36.3 | 24.5 |
| MUFA/PUFA Ratio | 1.19 | 2.66 | 2.70 | 2.14 | 1.25 |
| MUFA/SFA Ratio | 1.12 | 1.39 | 1.05 | 0.93 | 1.42 |


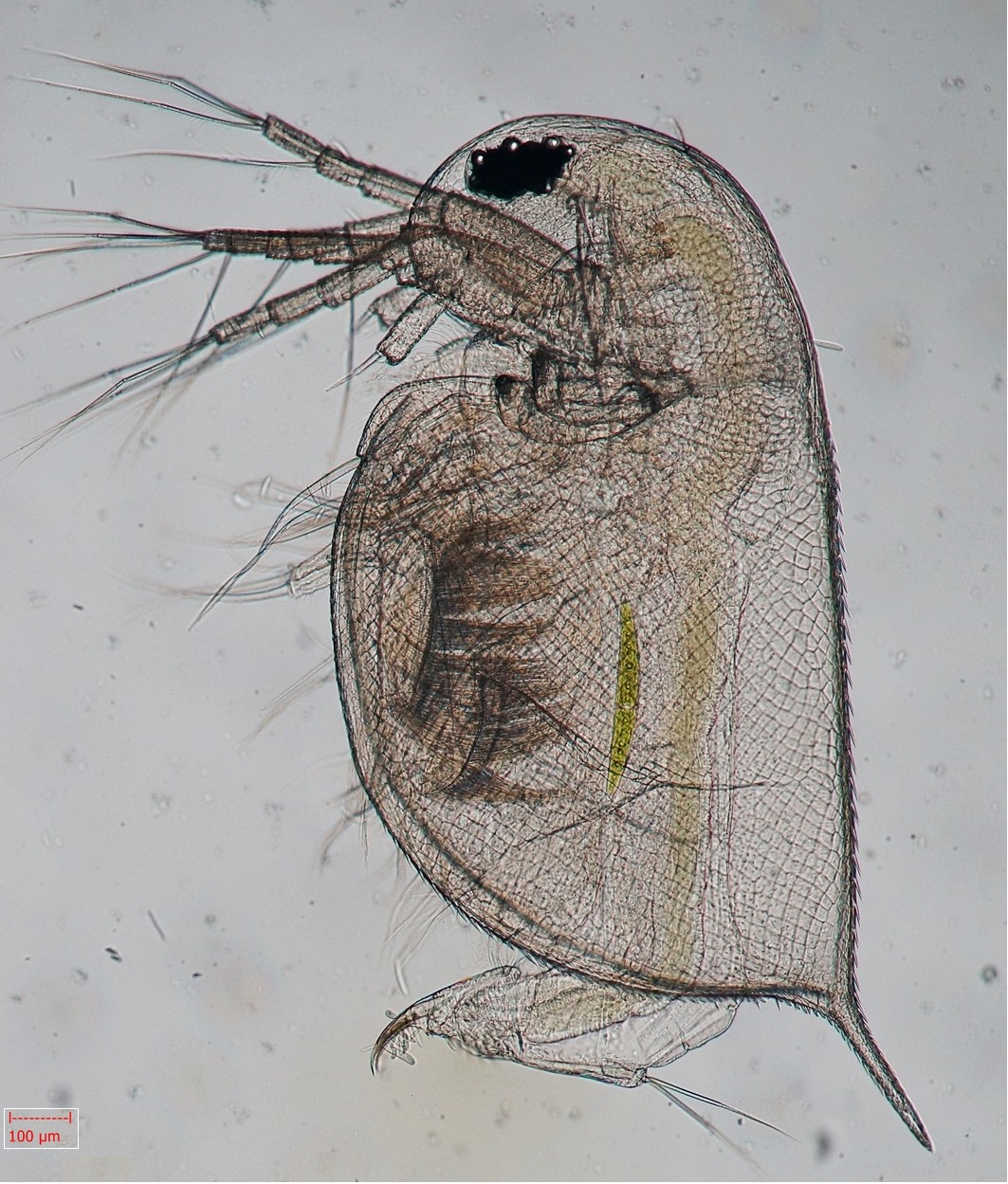
**Fig. S1.** A representative photomicrograph of *Daphnia* sp. isolated from HRAP and co-cultivated with isolated microalgal strains. This individual photographed from HRAP sample had ingested *Closterium* sp.
